# Moderate energy restriction with high protein diet results in healthier outcome in women

**DOI:** 10.1186/1550-2783-7-4

**Published:** 2010-01-25

**Authors:** Antti A Mero, Heikki Huovinen, Olle Matintupa, Juha J Hulmi, Risto Puurtinen, Hannele Hohtari, Tuomo AM Karila

**Affiliations:** 1 Department of Biology of Physical Activity, University of Jyväskylä, Jyväskylä, Finland; 2 Hospital Jorvi, Espoo, Finland; 3 Hospital Orton, Helsinki, Finland; 4 Orthopaedic Department, Gisborne Hospital, New Zealand

## Abstract

**Background:**

The present study compares two different weight reduction regimens both with a moderately high protein intake on body composition, serum hormone concentration and strength performance in non-competitive female athletes.

**Methods:**

Fifteen normal weighted women involved in recreational resistance training and aerobic training were recruited for the study (age 28.5 ± 6.3 yr, height 167.0 ± 7.0 cm, body mass 66.3 ± 4.2 kg, body mass index 23.8 ± 1.8, mean ± SD). They were randomized into two groups. The 1 KG group (n = 8; energy deficit 1100 kcal/day) was supervised to reduce body weight by 1 kg per week and the 0.5 KG group (n = 7; energy deficit 550 kcal/day) by 0.5 kg per week, respectively. In both groups protein intake was kept at least 1.4 g/kg body weight/day and the weight reduction lasted four weeks. At the beginning of the study the energy need was calculated using food and training diaries. The same measurements were done before and after the 4-week weight reduction period including total body composition (DXA), serum hormone concentrations, jumping ability and strength measurements

**Results:**

During the 4-week weight reduction period there were no changes in lean body mass and bone mass, but total body mass, fat mass and fat percentage decreased significantly in both groups. The changes were greater in the 1 KG group than in the 0.5 KG group in total body mass (p < 0.001), fat mass (p < 0.001) and fat percentage (p < 0.01). Serum testosterone concentration decreased significantly from 1.8 ± 1.0 to 1.4 ± 0.9 nmol/l (p < 0.01) in 1 KG and the change was greater in 1 KG (30%, p < 0.001) than in 0.5 KG (3%). On the other hand, SHBG increased significantly in 1 KG from 63.4 ± 17.7 to 82.4 ± 33.0 nmol/l (p < 0.05) during the weight reducing regimen. After the 4-week period there were no changes in strength performance in 0.5 KG group, however in 1 KG maximal strength in bench press decreased (p < 0.05) while endurance strength in squat and counter movement jump improved (p < 0.05)

**Conclusion:**

It is concluded that a weight reduction by 0.5 kg per week with ~1.4 g protein/kg body weight/day can be recommended to normal weighted, physically active women instead of a larger (e.g. 1 kg per week) weight reduction because the latter may lead to a catabolic state. Vertical jumping performance is improved when fat mass and body weight decrease. Thus a moderate weight reduction prior to a major event could be considered beneficial for normal built athletes in jumping events.

## Background

Diet and exercise are key elements in weight control. Numerous studies demonstrate successful body weight reduction following energy restriction and increased physical activity in obese male and female subjects [1-3]. Also non-obese people may benefit from moderate weight reduction, especially in sports such as weight lifting and wrestling but also in jumping events (e.g. high jump, ski jump) and esthetic events (e.g. gymnastics, dancing). In elite male wrestlers, it was observed that two to three weeks of vigorous weight reduction regimen before competition resulted in a marked loss in body weight (8%), in fat mass (16%), in lean body mass (8%) and also significant 63% decrease in serum testosterone [4]. In obese females a decrease in serum testosterone concentration during the weight reduction period [5,6] was also observed. A high protein diet is important during weight reduction to hinder lean tissue loss and to focus weight reduction on fat mass [7]. In high-protein diets, weight loss is initially high due to fluid loss related to reduced carbohydrate (CHO) intake, overall caloric restriction, and ketosis-induced appetite suppression. Beneficial effects on blood lipids and insulin resistance may often be due to the weight loss, not directly to the change in caloric composition. Very high-protein diets with limited CHO and fat for long periods may not be recommended because they may not provide the variety of foods needed to adequately meet nutritional needs, particularly regarding the intake of essential nutrients (e.g. vitamins and minerals) [8].

It is well established that the utilization of ingested nutrients for energy is inversely related to the thermogenesis of food. This is a phenomenon associated with the energy cost of nutrient absorption, processing and storage [9]. The loss of energy is highest for protein consisting of a 25-30% loss of the ingested energy, followed by CHO with a 6-8% loss and fat with only a 2-3% loss [10,11]. Consequently, a higher thermogenic response following the intake of protein compared to CHO and fat may make some contribution to weight reduction.

Therefore, the purpose of the present study was to examine the effects of a 4-week weight reduction comparing two different energy deficit diets with a moderately high protein intake on body composition, hormone concentration and strength performance in physically active normal weighted women. According to the literature there are no previous studies conducted with these settings in normally built non-competitive female athletes.

## Methods

### Subjects

Healthy normal weighted young women were recruited for the study that had at least six months history of recreational resistance and aerobic training. The suitability of the volunteers was determined with a questionnaire. The subject was excluded if she was a competitive athlete or she self-reported anorexia nervosa, coronary heart disease, an irregular menstrual cycle or administration of hormonal contraceptives during the last six months. The study was approved by the local University Ethics Committee and the accepted participants (n = 15) signed a written consent.

### Study design

**Figure 1:**
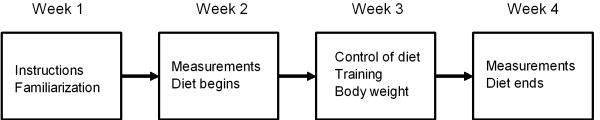
**Study design**.

At the beginning of the study the subjects were randomized to two groups: group 1 KG n = 8; age 28.0 ± 6.4 yr, height 167.0 ± 6.9 cm, body mass 66.9 ± 4.3 kg, body mass index 24.0 ± 1.5, and group 0.5 KG n = 7; age 28.9 ± 6.2 yr, height 167.0 ± 7.1 cm, body mass 65.7 ± 4.0 kg, body mass index 23.6 ± 2.0; mean ± SD. The group 1 KG (energy deficit 1100 kcal/day) was supervised to reduce body weight by 1 kg per week and the group 0.5 KG (energy deficit 550 kcal/day) by 0.5 kg per week during four weeks, respectively. Vitamin and mineral supplements (but not other e.g. sport drinks, creatine) were allowed and instructed to be used during the study period. Study design is shown in Figure 1.

### Instructions, Familiarization and Weight Reduction

One week before the beginning of the four week diet the subjects had a familiarization session with the exercises used in the strength tests and received general instructions for the study. The subjects kept food and training diaries during the next four days. The food diaries were analyzed using the Micro Nutrica nutrient-analysis software (version 3.11, Social Insurance Institution of Finland). The total energy requirement for each subject was evaluated by calculating resting metabolic rate (RMR) using the Owen formula (RMR = 795 + 7.18 × weight), which has been shown to be the best equation for normal-weighted women undergoing energy restriction [12,13]. Energy consumed in physical activity was estimated using the internet application EnergyNet (University of Kuopio). The total energy need was 2340 ± 170 kcal for 1 KG and 2290 ± 120 kcal for 0.5 KG. Energy deficit and diet for each subject during the weight reduction period was then evaluated. The group 1 KG (energy deficit 1100 kcal/day, protein at least 1.4 g/kg/day) was supervised to reduce body weight by 1 kg per week and the group 0.5 KG (energy deficit 550 kcal/day, protein goal at least 1.4 g/kg/day) by 0.5 kg per week during the next four weeks, respectively. The subjects kept food diaries for two days each week and the researchers could then, with the diaries and with morning scale body weights, supervise that body weight was reducing as planned. All subjects were advised to continue their normal recreational resistance training and aerobic training during the weight reduction period which was also controlled each week.

### Measurements

#### Body composition

Body scale weight was determined in the familiarization session, in the before and after measurements and in every week control with the same electric digital scale. Total body composition was determined using a dual-energy X-ray absorptiometry device (DXA; Lunar Prodigy Densitometer, GE Lunar Corporation, Madison, WI, USA). This method can differentiate bone mineral density (BMD), total percentage fat, total body tissue mass, fat mass, lean mass, bone mineral content (BMC), and total bone calcium with precision errors of 0.62, 1.89, 0.63, 2.0, 1.11, 1.10, and 1.09%, respectively [14].

#### Blood sampling and hormone analysis

Blood samples were drawn from the antecubital vein for analyze of hemoglobin, serum total testosterone, sex-hormone-binding globulin (SHBG), cortisol and dehydro-epiandrosterone sulfate (DHEAS) and pH were drawn on the morning of both measurement days after a 12 h fast. The intervention time interval was exactly 4 weeks for everyone so the menstrual cycle was in the same phase. The samples were taken in the sitting position two times with 30 minutes in between measurements. Serum samples were kept frozen at -80°C until assayed.

Two milliliters of blood was taken in K2 EDTA tubes (Terumo Medical Co., Leuven, Belgium) for measurements of hemoglobin concentration with a Sysmex KX 21N Analyzer (Sysmex Co., Kobe, Japan). The intra-assay coefficient of variation (CV) is 1.5% for hemoglobin.

For the determination of serum hormone concentrations five milliliters of blood was taken and the concentrations were analyzed by an immunometric chemiluminescence method with Immulite^® ^1000 (DPC, Los Angeles, USA). The sensitivity of the assay for serum testosterone is 0.5 nmol/l, for SHBG 0.2 nmol/l, for cortisol 5.5 nmol/l and for DHEAS 0.08 μmol/l. Coefficient of variations are 8.3% for testosterone, 5.0% for SHBG, 6.1% for cortisol and 8.8% for DHEAS. Free testosterone was calculated from total testosterone and immunoassayed SHBG concentrations [15,16].

pH was analyzed with Nova Biomedical STAT Profile pHOX Plus L Blood Gas Analyzator (Nova Biomedical, Waltham, MA, USA). The intra-assay CV is 0.1% for pH. All results are presented as the mean value of two samples described earlier.

#### Strength tests

Maximum strength (1RM) was measured in bench press with a free barbell and in full squat using a Smith machine. Strength endurance was measured performing as many repetitions as possible using a 50% load of 1RM in both bench press and in full squat. Jumping ability was measured using a counter movement jump (CMJ) on a contact mat with a clock [17]. The test order was as follows: CMJ, bench press 1RM, bench press strength endurance, full squat 1RM, and full squat strength endurance. Recoveries between trials were from three to five minutes in each test and at least five minutes between different tests. Continuous verbal encouragement was given during all test performances.

### Training

The subjects kept training diaries during the 4-week study period and they were analyzed every week in order to be sure that the subjects continued their individual normal recreational aerobic and resistance training.

### General mood

The subjects completed a 5-point Likert-like scale questionnaire at the end of the weight loss regimen. The questionnaire consisted of questions on alertness, general mood and self-confidence.

### Statistical Analyses

The independent t-tests, the Pearson's correlation coefficients and a regression analysis were used for statistical analysis and p ≤ 0.05 value was considered statistically significant.

## Results

### Energy intake

Both energy intake and protein intake were similar in the groups during the 4-week weight reduction period (average of eight days) and were 1330 ± 176 kcal and 99 ± 21 g (~1.5 g/kg body weight/day) in the 0.5 KG group and 1036 ± 234 kcal and 91 ± 17 g (~1.4 g/kg body weight/day) in the1 KG group, respectively. Also carbohydrate and fat intake were similar in the groups (carbohydrates 156 ± 25 g in 0.5 KG and 115 ± 35 g in 1 KG, fat 33 ± 5 g in 0.5 KG and 23 ± 20 g in 1 KG).

### Hemoglobin

Hemoglobin was 124 ± 7 g/l and 127 ± 5 g/l in 0.5 KG before and after the 4-week period. The respective concentrations in 1 KG were 130 ± 11 g/l and 134 ± 7 g/l. There were no significant differences between the groups.

### pH

After the 4-week weight reduction period pH increased from 7.43 ± 0.04 to 7.48 ± 0.03 (p = 0.05) in 0.5 KG and in 1 KG from 7.44 ± 0.03 to 7.46 ± 0.04 (p = 0.19). The difference between the groups did not reach statistical significance (p = 0.23).

### Training

The groups trained similarly. During the 4-week period the average training for all subjects per week was as follows: 90 minutes resistance training (body pump, circuit, weight training), 110 minutes aerobic training (running, cycling, aerobics) and very low aerobic training (brisk walking, vacuuming) for 280 minutes.

### Body composition

**Figure 2:**
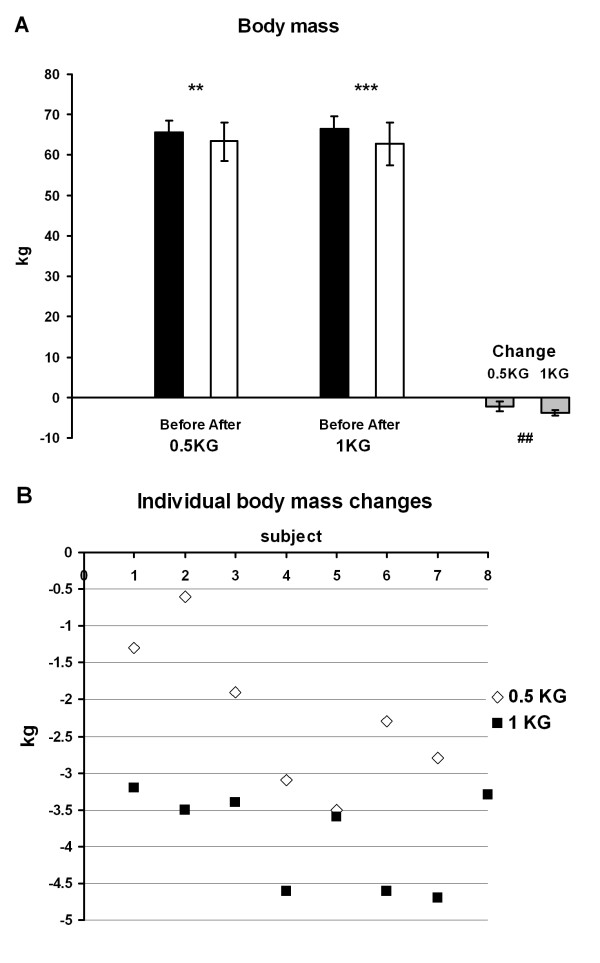
**a -- The body mass and the change of the body mass in both groups before and after the 4-week weight reduction**. ## p < 0.01, ** p < 0.01, *** p < 0.001. **b**-The individual body mass changes during the 4-week weight reduction period in the 0.5 KG and 1 KG groups.

**Figure 3:**
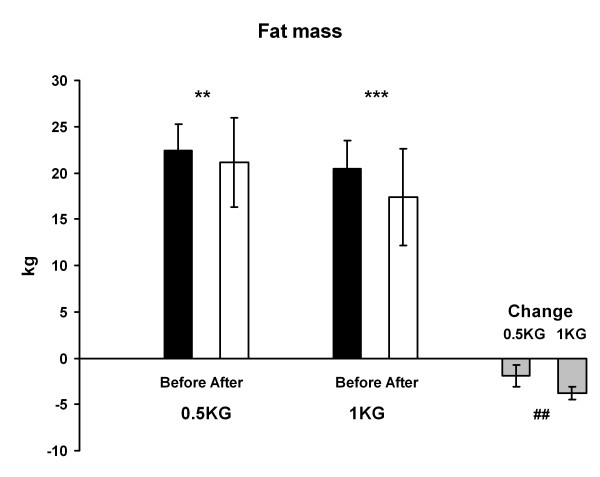
**The fat mass and the change of the fat mass in both groups before and after the 4-week weight reduction**. ## p < 0.01 difference between the groups in the change from before to after situation, ** p < 0.01, *** p < 0.001 difference from before to after situation.

Total body mass (Figure 2a, b) and fat mass (Figure 3) decreased in the 1 KG group (p < 0.001) and in the 0.5 KG group (p < 0.01). The change was greater in 1 KG than in 0.5 KG in both cases (p < 0.01). There were no changes in lean body mass or bone mass.

### Hormone concentrations

**Figure 4:**
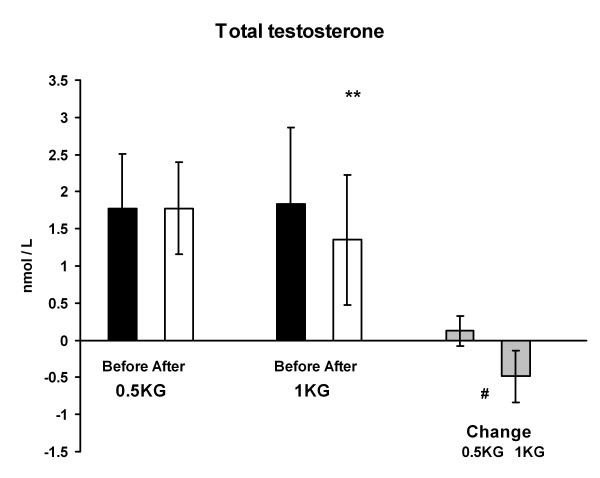
**The serum total testosterone concentration and the change of it after the 4-week weight reduction in both groups**. # p < 0.05 difference between the groups in the change from before to after situation, ** p < 0.01 difference from before to after situation.

**Figure 5:**
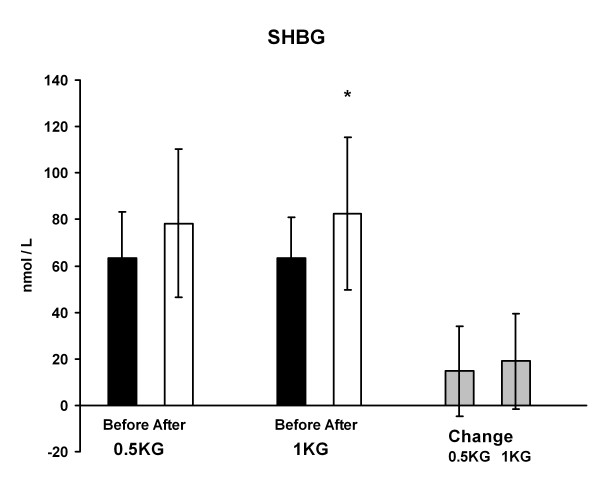
**The SHBG concentration and the change of it after the 4-week weight reduction in both groups**. * p < 0.05 difference from before to after situation.

**Figure 6:**
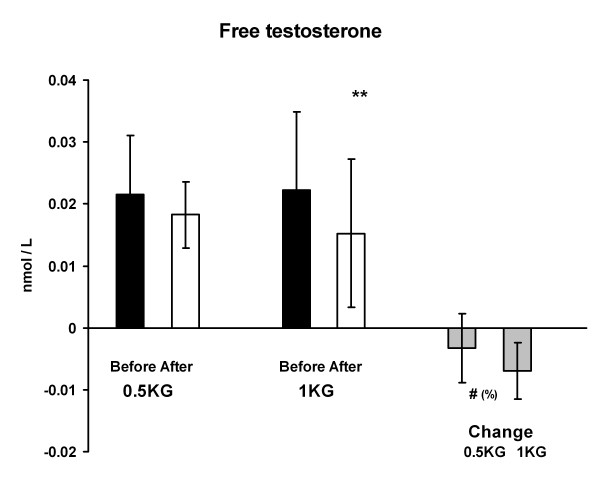
**The serum free testosterone concentration and the change of it after the 4-week weight reduction in both groups**. ** p < 0.01 difference from before to after situation, # p < 0.05 relative change (%) between the groups.

Serum total testosterone concentration decreased significantly from 1.8 ± 1.0 to 1.4 ± 0.9 nmol/l (p < 0.01) in 1 KG and the change was greater (p < 0.05) in 1 KG than in 0.5 KG (Figure 4). On the other hand, serum SHBG concentration increased in 1 KG from 63.4 ± 17.7 to 82.4 ± 33.0 nmol/l (p < 0.05) during the weight reducing regimen. The change in the 0.5 KG group did not reach the level of statistical significance (Figure 5). Serum free testosterone decreased significantly only in 1 KG (p < 0.01) and the change was relatively greater (p < 0.05) in 1 KG than in 0.5 KG (Figure 6). There were no differences in serum cortisol or DHEAS concentration within or between the groups. The cortisol concentration was 577 ± 162 nmol/l in 0.5 KG and 496 ± 183 nmol/l in 1.0 KG before the weight loss. After the weight loss the concentration was 581 ± 205 nmol/l in 0.5 KG and 568 ± 170 nmol/l in 1.0 KG. The DHEAS concentration was 4.8 ± 2.4 μmol/l in 0.5 KG and 5.4 ± 5.0 μmol/l in 1.0 KG before the period. After the weight loss the concentration was 4.9 ± 2,3 μmol/l in 0.5 KG and 5.6 ± 3.0 μmol/l in 1.0 KG.

### Correlations

**Figure 7:**
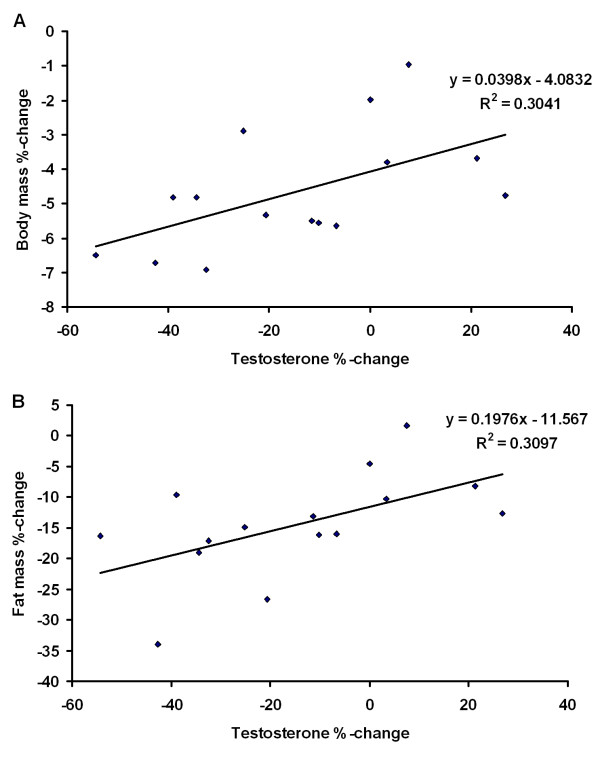
**The relationships between the percentage change in the serum testosterone concentration and the percentage changes in total body mass (above A) and fat mass (below B)**.

The percentage change in serum testosterone concentration correlated significantly with the percentage change in body mass (r = 0.55, p = 0.033) and with the percentage change in fat mass (r = 0.52, p = .045). Also regression equations are shown (Figure 7A and 7B).

### Strength performance and jumping ability

There were no differences in performance changes between 1 KG and 0.5 KG after the 4-week period but in 1 KG maximal strength in bench press decreased (p < 0.05) and CMJ improved (p < 0.02) (Table 1).

**Table 1:** Characteristics of physical performance (mean ± SD)

Variable	Before	After	Before vs. after (p =)	Sign. in change 0.5 KG vs. 1 KG (p =)
Bench press (kg) 1RM 0.5 KG	31.1 ± 8.8	31.1 ± 8.8	1.00	0.10
Bench press (kg) 1RM 1 KG	36.3 ± 7.1	34.7 ± 6.3	0.05	
Bench press ME 0.5 KG(reps × kg)	502 ± 200	481 ± 190	0.35	0.44
Bench press ME 1 KG (reps × kg)	657 ± 175	661 ± 203	0.87	
Squat 1RM (kg) 0.5 KG	61.8 ± 24,1	63.9 ± 24,5	0.25	0.49
Squat 1RM (kg) 1 KG	58.8 ± 13.6	59.7 ± 14.6	0.20	
Squat ME 0.5 KG (reps × kg)	991 ± 545	1003 ± 556	0.93	0.16
Squat ME 1 KG (reps × kg)	1460 ± 1076	1956 ± 1733	0.11	
CMJ 0.5 KG (cm)	43.7 ± 5,9	45.0 ± 6.7	0.12	0.75
CMJ 1 KG (cm)	46.0 ± 2,4	47.0 ± 3.0	0.02	

Data are means ± SDs. 1RM = one repetition maximum, ME = muscle endurance (repetitions × load), CMJ = counter-movement jump

### General mood

In 0.5 KG, 57% of the subjects (n = 4/7 = 4 subjects from 7 subjects) reported that they had more alertness in work/studying and training during the weight loss regimen. Similarly in 1.0 KG, 44% of the subjects (n = 3/8) reported that they had more alertness in school and only 25% reported that they had more alertness during training. Furthermore in 1.0 KG, 50% of the subjects (n = 4/8) reported that they had felt less alertness during training when no one in 0.5 KG gave such an answer (n = 0/7). The subjects in 0.5 KG also reported better general mood and no one from this group reported any kind of anxiety when 37.5% (n = 3/8) in 1.0 KG reported that they were more anxious and felt more tired than usual. Almost everyone in both groups was satisfied with the weight loss and thought that they looked better after the weight loss (n = 14/15).

## Discussion

### Main results

We were able to demonstrate significant changes in body composition after a 4-week weight reduction regimen as total body weight, fat mass and fat percentage decreased in both groups. The changes were significantly greater in the 1 KG group than in the 0.5 KG group. Serum total and free testosterone concentrations decreased significantly in 1 KG, though the change was greater in 1 KG than in 0.5 KG. On the other hand, SHBG increased significantly in 1 KG group during the weight reduction regimen. After the 4-week period there were no changes in strength performance in 0.5 KG but in 1 KG maximal strength in bench press decreased whereas endurance strength in squat and CMJ improved.

### Diet composition and body composition

We were successful in diet intervention in both groups in decreasing carbohydrates and fat and in increasing protein intake as calculated from the 8-day food records during four weeks. The daily amounts of carbohydrates (115-156 g) and fat (23-33 g) consumed were very small and the protein intake was moderately high in both groups (91-99 g). The total energy need calculated at the beginning of the study was 2340 kcal for 1 KG group and 2290 kcal for 0.5 KG group and during the weight reduction period energy intake was 1036 kcal for 1 KG and 1330 kcal for 0.5 KG. Healthcare professionals have suggested that women should have a minimum of 1200 kcal per day during a weight reduction period which means that our 1 KG group was slightly below the limit [8]. Consequently, it means that caloric restriction was 56% in the 1 KG and 42% in the 0.5 KG group which resulted in body weight decreases of 4.6 kg and 2.5 kg, respectively. Although it should be noted that enough essential fatty acids, vitamins and minerals were planned to be contained in each diet it is possible that some subjects were undernourished in these nutrients even though they were advised to use vitamin and mineral supplements. This should be taken into account when planning longer lasting weight reduction programs [8].

Hemoglobin concentration remained the same in both groups during the study although there might be some fluid decrease induced by the diet. Blood pH increased in both groups but only significantly in the 0.5 KG group (from 7.43 to 7.48). This could be explained by markedly decreased carbohydrate intake (especially sugar and wheat) and increased intake of fruits and vegetables which could lead to an enhanced amount of bases [18], although the amount of protein consumed (acidotic) was quite high (1.4 g/kg body weight/day). Metabolic acidosis has been linked to muscle wasting in obese subjects who were acidotic due to weight reduction diets [19,20]. The correction of the acidosis has been shown to reverse the muscle wasting in that condition [21,22]. According to a recent study by Dawson-Hughes et al. [23], higher intake of foods rich in potassium, such as fruit and vegetables, may favor the preservation of muscle mass in older men and women. In the present study muscle mass was remained the same during the study and the elevated pH was probably due to that.

The present results show that weight reduction with a high protein diet markedly decreased fat mass in both groups (-2.0 kg in 0.5 KG and -3.8 kg in 1 KG) which is concordant with findings reported by Layman et al. [7]. Their daily diet regimen included less than 150 g carbohydrates and protein over 1.4 g/kg. The fat decrease in our normal weighted women was almost the same as the total decrease in body weight. A small part of the weight reduction is probably due to decreased body fluids, because weight loss is initially high due to fluid loss related to reduced carbohydrate intake, reduced muscle glycogen concentration, overall caloric restriction, and ketosis-induced appetite suppression. On the other hand, it was somewhat surprising that lean body mass remained constant during the 4-week period in both groups. This may indicate that this kind of diet combination with 1.4 g protein intake daily is efficient in decreasing fat without major loss of muscle mass. High-protein diets are considered to be more effective in weight reduction and weight maintenance than other diets (such as higher carbohydrates-to-protein or fat-to-protein ratios). Not only because of the higher energy cost of nutrient absorption, processing and storage [9], but also due to their high-satiating and thermogenic effect [24-30]. Recently Claessens et al. [31] concluded that high-casein or whey protein with low-fat diet weight maintenance is more effective than low-fat, high-carbohydrate diets. Also high-casein or whey protein with low-fat diet does not adversely affect on metabolism or increase cardiovascular risk in moderately obese subjects. Our study is concordant with these findings in young healthy normal weighted women.

### Hormone concentrations

In the 1 KG group there was a significant decrease (30%) in serum testosterone concentration and an increase in SHBG which causes a decrease in free testosterone. Approximately 65% of serum testosterone is bound to SHBG [32]. Another portion is bound to albumin (about 33%). The free testosterone is considered the active fraction and represents approximately 2% of total testosterone [32]. Consequently, bioavailable free testosterone levels have been inversely related to the levels of SHBG [33]. It has been known for a long time that obese women have high testosterone concentrations [34] and in them weight reduction resulted in lowered testosterone concentrations [5]. We were also able to demonstrate a significant correlation between decreased serum testosterone concentration and the amount of lost body and fat weight. That finding is concordant with a finding of Karila et al. [4] in men.

In the study by Pasquali et al. [6] serum testosterone concentrations decreased significantly in obese women during eight months when their body mass decreased on average only 1.35 kg per month. In the present study with normal weighted subjects the intervention lasted only 4 weeks and it is possible that the testosterone concentration would have decreased significantly in the 0.5 KG group as well if the intervention had lasted for a longer time. It is obvious that the 4-wk period with a moderately lowered serum testosterone concentration was too short to cause a catabolic state which could be noted as markedly decreased lean body mass. On the other hand it might have negatively affected muscle mass if the weight reduction diet would have been prolonged. The SHBG concentration increased in both groups but significantly (28%) only in the 1 KG group. It was expected because significant weight loss obtained through reduced caloric intake leads to increased SHBG concentration regardless of diet composition, particularly in women [35,36]. In the present study there were no changes in serum DHEA and cortisol concentrations and their role has been unclear in diet interventions [36].

### Physical performance

The changes between the groups were not significant in physical performance but vertical jumping performance improved in 1 KG and tended to also improve in 05 KG as expected [37]. This could be mainly due to decreased fat and body weight. Thus in competitive female athletes moderate weight reduction prior to a major competition (e.g. in jumping events) could be encouraged in order to perform better. In the same 1 KG group the decrease in maximal bench press was also somewhat expected with markedly lowered body mass but in 0.5 KG the decrease was only slight.

### General mood

It seems that the subjects with 0.5 kg weight reduction felt somewhat fresher at work, at school and in training compared to the other subjects. On the other hand, the subjects with more weight reduction were more satisfied with their body image and felt better about themselves. Consequently, general mood was quite similar in the groups. Earlier [38] it has been discussed that weight reduction may have positive effects on depression.

## Conclusion

It is concluded that a weight reduction of 0.5 kg per week with ~1.4 g protein/kg/day can be recommended to normal weighted, physically active women instead of a larger (e.g. 1 kg per week) weight reduction, because the latter may lead to a catabolic hormonal state in the body after four weeks. Vertical jumping performance will be improved when fat mass and body weight decrease and thus weight reduction before an important competition (e.g. in jumping events) could be encouraged. Nevertheless, further studies with athletes are needed in order to verify this hypothesis.

## Competing interests

The authors declare that they have no competing interests.

## Authors' contributions

AAM conceived the study, developed the study design, participated in data acquisition and drafting the manuscript. HHu and OM developed the study design, participated in the data acquisition and assisted in drafting the manuscript. HHu and OM designed the diets and supervised the subjects during the weight reduction period. JJH assisted with the design of the study and the manuscript preparation. RP collected blood samples and analyzed them. HHo and TAMK assisted with the design of the study and drafting the manuscript. All authors have read and approved the final manuscript.
